# Epigenetic Mechanisms of ART-Related Imprinting Disorders: Lessons From iPSC and Mouse Models

**DOI:** 10.3390/genes12111704

**Published:** 2021-10-26

**Authors:** Alex Horánszky, Jessica L. Becker, Melinda Zana, Anne C. Ferguson-Smith, András Dinnyés

**Affiliations:** 1 BioTalentum Ltd., H-2100 Gödöllő, Hungary; alex.horanszky@biotalentum.hu (A.H.); melinda.zana@biotalentum.hu (M.Z.); 2 Department of Physiology and Animal Health, Institute of Physiology and Animal Health, Hungarian University of Agriculture and Life Sciences, H-2100 Gödöllő, Hungary; 3 Department of Genetics, University of Cambridge, Cambridge CB2 3EH, UK; jlb208@cam.ac.uk (J.L.B.); afsmith@gen.cam.ac.uk (A.C.F.-S.); 4 HCEMM-USZ Stem Cell Research Group, Hungarian Centre of Excellence for Molecular Medicine, H-6723 Szeged, Hungary; 5 Department of Cell Biology and Molecular Medicine, University of Szeged, H-6720 Szeged, Hungary

**Keywords:** genomic imprinting, imprinting disorders, assisted reproductive technology, DNA methylation, mouse models, iPSCs

## Abstract

The rising frequency of ART-conceived births is accompanied by the need for an improved understanding of the implications of ART on gametes and embryos. Increasing evidence from mouse models and human epidemiological data suggests that ART procedures may play a role in the pathophysiology of certain imprinting disorders (IDs), including Beckwith-Wiedemann syndrome, Silver-Russell syndrome, Prader-Willi syndrome, and Angelman syndrome. The underlying molecular basis of this association, however, requires further elucidation. In this review, we discuss the epigenetic and imprinting alterations of in vivo mouse models and human iPSC models of ART. Mouse models have demonstrated aberrant regulation of imprinted genes involved with ART-related IDs. In the past decade, iPSC technology has provided a platform for patient-specific cellular models of culture-associated perturbed imprinting. However, despite ongoing efforts, a deeper understanding of the susceptibility of iPSCs to epigenetic perturbation is required if they are to be reliably used for modelling ART-associated IDs. Comparing the patterns of susceptibility of imprinted genes in mouse models and IPSCs in culture improves the current understanding of the underlying mechanisms of ART-linked IDs with implications for our understanding of the influence of environmental factors such as culture and hormone treatments on epigenetically important regions of the genome such as imprints.

## 1. Background

The epigenetic process of genomic imprinting regulates the expression of a subset of genes in a parent-of-origin specific manner. Through this mechanism, only the maternal or paternal allele of an imprinted gene is expressed, while the other allele is epigenetically repressed [1]. *Cis*-acting regulatory elements called imprinting control regions (ICRs) confer imprinting on neighbouring genes. During male and female germline development, de novo methyltransferases methylate ICRs in a parental-specific fashion and these marks withstand post-fertilization epigenetic reprogramming to act as a memory of parental origin [2]. Hence imprinting is regulated by germline derived differential methylation that persists after fertilisation resulting in monoallelic gene expression and the correct dosage of imprinted gene products during development. Imprinted prenatal development and resource provisioning occur in the placenta and foetal growth, as well as in postnatal energy homeostasis, brain function, and behaviour [3,4,5]. Therefore, the proper establishment and maintenance of epigenetic control of the imprinted genes are pivotal to both the development of the conceptus and postnatal health.

The influence of multiple imprinted genes has been further elucidated through studies of patients exhibiting diseases now known as imprinting disorders (IDs). There are at least a dozen diseases that can be classified as IDs, and many share similar phenotypes which can make diagnosis difficult [6]. Aberrant pre-/postnatal development, hormone imbalances, learning and behavioural impairments, and/or poor feeding behaviour have been identified as common clinical features of patients with IDs. Furthermore, different IDs can involve the same imprinted locus depending on the parental origin of the molecular disruption. Copy number variation, uniparental disomy (UDP), epimutations, and genetic mutations are the four molecular defects that have been linked to IDs. The defects are not mutually exclusive, as a genetic mutation at a modifier locus may lead to epimutations elsewhere.

In recent years, an increasing number of reports have suggested a relationship between assisted reproductive technology (ART) and IDs. Multiple studies examining different cohorts have noted an increased rate of Beckwith-Wiedemann syndrome (BWS), Angelman syndrome (AS), Prader-Willi syndrome (PWS), and Silver-Russell syndrome (SRS) in ART populations [7,8,9,10,11,12]. While it is plausible that ART may interfere with the establishment and/or maintenance of imprints, the data are not comprehensive enough to draw definitive conclusions. Much of the patient data are incomplete and lack molecular characterisations of the diagnoses. Additionally, factors such as infertility, maternal age, and specific ART methods are often not included in the analyses. Nevertheless, as the frequency of ART-facilitated births proceeds to increase, so does the importance of understanding the effects of ART on gametes and embryos.

## 2. Imprinting Disorders Associated with ART

Understanding the molecular bases underlying the ID in ART patients may reveal patterns of vulnerability associated with ART procedures. Because no single molecular aberration defines an ID, if ART populations show enrichment for a particular defect, we may be able to determine modes of susceptibility. This can help improve ART technologies while also expanding our understanding of imprinting and the susceptibility of imprints to environmental influence more generally.

### 2.1. Beckwith-Wiedemann Syndrome

BWS is classified as an overgrowth disorder and predisposes the individual to cancerous and noncancerous tumour growth. The molecular changes most associated with BWS affect the chromosome 11p15.5–11p15.4 region which includes two closely linked clusters of imprinted genes and two ICRs. The paternally expressed insulin-like growth factor 2-encoding gene (*IGF2*) and the maternally expressed long non-coding RNA (lncRNA) *H19* are controlled by the *H19/IGF2:IG-DMR*, while the maternally expressed cell cycle inhibitor gene *CDKN1C*, neighbouring imprinted genes, and the paternally expressed lncRNA *KCNQ1OT1* are controlled by the *KCNQ1OT1*:TSS-DMR. Epimutations are the most common molecular defect seen in BWS, as 50% of patients exhibit loss of methylation (LOM) at the maternal *KCNQ1OT1*:TSS-DMR and 5–10% show gain of methylation (GOM) at the maternal *IGF2/H19* DMR [13]. However, UPD, *CDKN1C* mutations, and general chromosomal abnormalities have also been detected [14].

The prevalence of BWS in naturally conceived children is estimated to range from 1 in 13,700 to 1 in 287,000 live births [15]. Conversely in ART populations it has been demonstrated to be as high as 1 in 1126 live births in one USA-based study [16], although varying results have been described in other countries, with some reporting no association between ART and BWS [17].

In a study using BWS patients from the Spanish Overgrowth Syndrome Registry [18], 51% of spontaneously conceived individuals with BWS (71/139) exhibited epimutations, with 13% showing UPD, 11% with *CDK1NC* mutations, and 6.5% with chromosomal rearrangements. Comparatively, 88% of the ART cohort (15/17) showed *KCNQ1OT1*:TSS-DMR hypomethylation. However, the ART cohort only accounted for 9% of the total population assessed (17/187). Another study found no significant differences in the frequency of *KCNQ1OT1*:TSS-DMR hypomethylation among the ART (15/16) and non-ART (21/24) BWS cohorts [19]. Meta-analyses suggest an overrepresentation of *KCNQ1OT1*:TSS-DMR LOM in ART populations, though all the included studies remain hindered by low sample sizes [18]. 

### 2.2. Silver-Russell Syndrome

SRS is associated with intrauterine growth restriction, low birth weight, slow postnatal growth, and body asymmetry. Diagnosis of SRS is therefore particularly difficult, as many clinical features of the disease are non-specific and the underlying molecular cause can only be identified in 60% of patients [20]. The most common mechanism, reported in 30–60% of patients, is LOM at the *H19/IGF2:IG*-DMR in the 11p15.5 region [21]. Alternatively, the maternal UPD of chromosome 7 is seen in 5–10% of the SRS population, however there is no consistent pattern in which the chromosome 7 imprinted genes are disrupted in SRS patients [22]. *MEST* and *GRB10* are known imprinted genes found on chromosome 7, yet sequencing and methylation studies of patients suggest that these genes are not perturbed in SRS.

The incidence of SRS in the naturally conceived population is estimated to range from 1 in 30,000 to 1 in 100,000 [21], however the literature is lacking in reliable estimates regarding SRS incidence in ART populations. Two epidemiological studies in Japan conducted in 2009 and 2015 found that all ART-SRS patients sampled had DNA methylation errors, while the non-ART cohort demonstrated the expected distribution of UPD and methylation errors [23,24]. SRS was the ID with the highest frequency in the 2015 survey, with the number of ART-SRS patients 8.91-fold higher than expected (8/67). However, as with the BWS studies, the ART cohort sizes are significantly smaller than those of the non-ART. Furthermore, no novel imprinting errors were found within the ART-SRS population. Given the currently available patient data and general lack of understanding of SRS, one cannot assert a strong correlation between ART and this ID. 

### 2.3. Prader-Willi Syndrome

Individuals with PWS exhibit a broad range of symptoms, including restricted growth, learning difficulties, and hypotonia. Both the genomic and epigenetic changes that cause PWS affect the paternally expressed genes on chromosome 15q11.2-q13. Microdeletion of the paternal copy of chromosome 15 accounts for the underlying defect in 65–75% of the PWS population, with maternal UDP seen in 20–30% and epimutations found in <5% of patients [25]. The affected imprinted genes are *MKRN3*, *MAGEL2*, *NECDIN*, and *SNURF-SNRPN*, as well as six small nucleolar RNAs (snoRNAs). 

The relationship between PWS and ART is the subject of debate, with recent studies offering contradicting conclusions. The prevalence of PWS in naturally conceived children is approximately 1 in 10,000 to 1 in 30,000 live births [15]. Analyses of Danish, Finnish, and American PWS cohorts did not find a significant increase in the rate of PWS among ART populations [7,26]. Another study reported a 1.5% incidence of PWS in ART patients (4/261), although this increase is not significant compared to naturally conceived children [27]. In contrast, the 2015 epidemiological study from Japan noted an association between ART and PWS [24]. Potential differences in regional methodologies and the numerous limitations to ART population studies can explain the conflicting results. However, even the studies that do not show an overall increase in PWS offer important insights to IDs in ART groups. Gold and colleagues found an increase in the rate of maternal UDP and methylation errors in PWS-ART patients [26]. Unfortunately, these two populations were grouped together and the source data did not distinguish the two mechanisms. Maternal age has been previously implicated in increased maternal UDP in PWS patients [28]. As advanced maternal age is enriched in ART populations, we cannot determine if this association is a result of the ART procedures or age of the mother. Nevertheless, the study shows that even if ART does not influence the frequency of IDs, it may cause novel epigenetic errors that lead to IDs. Detailed characterizations of the epigenomes of ART-conceived individuals, especially those with IDs, will improve our understanding of how ART procedures affect DNA modifications.

### 2.4. Angelman Syndrome

Characteristics of AS, which affects approximately 1 in 15,000 people [15], are developmental delays, intellectual disability, speech impairment, and ataxia. The molecular basis of AS is well-characterised as all defects affect the *UBE3A* gene on 15q11.2-q13, which is maternally expressed in the developing brain. The distribution of molecular defects of AS is like that of PWS, with many cases (65–75%) resulting from microdeletions on the maternal chromosome [29]. Unlike the previously mentioned IDs, recent studies have negated the association between AS and ART, with no significant increase in prevalence compared to naturally conceived births, and no novel molecular disruption is found in AS-ART patients [7,24,30]. However, models of AS may prove to be a useful tool in understanding the mechanisms of imprinting, given the clearly defined temporal, spatial, and chromosomal impacts of the disease.

## 3. A Need for Improved Imprinting Model Systems

The effects that ART procedures exert on the gamete and embryo have been the subject of many investigations in recent times using experimental systems. These procedures occur during developmental windows associated with a critical temporally coordinated period of epigenetic reprogramming that is vulnerable to epigenetic aberrations [31]. The potential to comprehensively assess ART-induced effects on DNA methylation and imprinting is limited by the heterogenous nature of fertility treatments, differences between imprinted regions, and the various tissues and techniques used for measurements [32]. Larger and more well-defined cohorts and a standardization of measurement techniques are required to overcome these complications. Mouse models of ART overcome many of the issues presented in the epidemiological human studies, while stem cells hold increasing promise for modelling imprinting disorders associated with ART.

ART procedures can include manipulations of different stages of the conception process, such as stimulating gamete generation and ex vivo embryonic cultures. Several processes involved in ART can potentially perturb normal genomic imprinting. Such processes include in vitro oocyte maturation, direct injection of sperm in ICSI, transferring in vitro cultured preimplantation embryos to the uterus, cryo-storage of embryos and gametes, and hormone induced downregulation of pituitary functions for superovulation [31]. Here, we review the epigenetic alterations and imprinting status in mouse models of ART and of iPSCs in culture, assessing their strengths and weaknesses as models for genomic imprinting and imprinting disorders associated with ART procedures. Combining the knowledge obtained from these models and comparing patterns of vulnerability in imprinted loci can allow an improved understanding of the underlying mechanisms of imprinting disorders associated with ART, which can in turn lead to the development of potential therapies and preventative measures.

## 4. Mouse Models for Imprinting and ART

Mouse models are an essential tool for the study of genomic imprinting and there is strong conservation of the mechanisms underlying imprinting in mouse and man [33]. The ICRs, genes, and epigenetic modifications that regulate gene expression in a parent-of-origin manner are mostly conserved between humans and mice. The functions of several imprinted genes, the regulation of key imprinted clusters, such *Cdkn1c/Kcnq1ot1* and *Igf2/H19*, and the effects of aberrant imprinting on gene function have been initially characterized in mice [33,34,35,36]. Animal models are also advantageous for investigations into the underlying mechanisms of IDs. Mouse models of several IDs, including those associated with ART, have been developed, including BWS, SRS, PWS, AS and KOS, and Temple syndromes [37,38,39,40,41].

While it is currently unclear which aspects of ART procedures may perturb imprinting in human populations, advancements have been made in mouse studies. Numerous groups have reported DNA methylation errors at imprints in oocytes, placentas, and embryos after superovulation procedures in mice [42,43,44,45,46,47,48]. One study even found that superovulation altered *H19* expression and *Grb10* methylation more severely than IVF or in vitro maturation [45]. The mouse models recapitulate human studies, which show that methylation of *H19*, *PEG1*, and *KCNQ1OT1* is also altered in human oocytes after superovulation [49,50,51]. The expression of ZFP57, a key regulator of mouse imprints post-fertilisation, is significantly reduced after superovulation in mouse oocytes [47]. However, in contrast to the mouse, ZNF57 is not detected in human oocytes; instead, ZNF445 is believed to confer the earliest methylation maintenance role at imprints post-fertilisation [52,53]. Nonetheless, the mouse studies suggest that superovulation has the potential to influence the expression of oocyte factors that regulate post-fertilisation methylation imprints, which could contribute to epimutation and lead to IDs. 

Other components of ART procedures have been examined individually in mice. Cryopreservation of mouse embryos has been shown to affect methylation at *Kv*DMR1 [54]. Another study found that the blastocysts and morulae of mice conceived via IVF displayed abnormal DNA methylation at the *Igf2*/*H19* imprinted locus [55]. Numerous other factors, such as culture media and the selection of fast-growing or slow-growing cultured embryos for implantation, have been shown to have epigenetic consequences at the imprinted loci [48,56]. The culture of mouse embryos has been demonstrated to dysregulate imprints, with LOI detected at the maternally imprinted *Peg3 and Snrpn*, and the paternally imprinted *H19*, although in the literature there seems to be no difference between the frequency of LOI in maternally and paternally methylated DMRs [57,58,59]. There are several postulated mechanisms for the aberrant imprinting displayed in cultured embryos, such as changes in the expression and subcellular localization of DNMTs that are critical for imprint maintenance [9]. 

Mouse studies have also been able to tease apart certain biases within human ART populations, including maternal age. While maternal age may influence chromosomal defects such as UPD, several studies have shown that it does not affect the methylation of imprints [60,61]. Table 1 summarizes many of the key findings of mouse studies of ART and imprinting.

Although mouse models have provided advancements in knowledge of genomic imprinting, there are limitations to modelling human imprinting defects in mice. For example, a causative factor of BWS, paternal UPD11, cannot be properly modelled using mice because uniparental disomy of mouse chromosome 7 causes embryonic lethality [69,70]. Moreover, paternal UPD11 patients display mosaicism, which has not been observed in mice [34]. Even subtle divergences in genetic and epigenetic regulation between mice and humans justify the need for human models of genomic imprinting.

## 5. iPSCs as a Tool to Model Imprinting Disorders

Due to the limitations presented by traditional mouse models, other strategies involving iPSCs can be utilized to study imprinting disorders and the effects of ART on imprinted gene regulation. The advantages of using iPSCs derived from individuals with IDs include the preservation of genotype associated with imprinting disorders, while their use eradicates the need to induce genetic mutations that could otherwise lead to off-target effects. Imprinting disorders can have complex and diverse aetiologies and therefore extensive engineering is required to generate the full representation of the associated genetic and epigenetic effects. ID-derived iPSCs offer a promising alternative as lineage-specific differentiation of iPSCs can also be used to further investigate the effects of imprinting disorders in various tissues that would be difficult to obtain from human patients. Combining knowledge derived from iPSCs and mouse models can enable further insight into the genetic/epigenetic mechanisms involved in ART-related imprinting disorders.

Stem cells have the exceptional capabilities of proliferation, self-renewal, and differentiation [71]. When given the correct conditions, self-renewing stem cells have the capacity to differentiate into virtually any cellular lineage, and are therefore an invaluable resource for disease modelling, the study of early human embryogenesis, and regenerative therapies [72]. The generation of iPSCs via the ectopic expression of reprogramming factors in adult somatic cells was ground-breaking and enabled the production of patient-specific, autologous iPSCs that pose no risk of immune rejection in cell-based therapies [73,74,75]. iPSCs share common features with ESCs, including development potential, proliferation capacity, morphology, and similar gene expression and epigenetic patterns [76,77,78].

The use of iPSCs is increasingly appealing for modelling conditions that involve intricate genetic abnormalities, including imprinting disorders. Epigenetic status is erased and reset during iPSC reprogramming and imprinted gene expression relies upon the successful maintenance of epigenetic signatures. Thus, a thorough analysis of allele-specific gene expression and imprinting status is critical when modelling disorders associated with genomic imprinting, to ensure that the disease-related epigenetic modifications are preserved in the obtained iPSCs. The successful production of iPSCs from patients with imprinting disorders such as AS and PWS was previously reported [79,80]. In one of these studies [79], Martins-Tyler and colleagues derived iPSCs from a PWS patient with a small, atypical deletion spanning the *SNORD116* cluster and *IPW* ncRNAs. It was shown that *UBE3A* displayed monoallelic expression and the lncRNA *UBE3A-ATS* was expressed in the obtained iPSCs. Assessment of the PWS-IC in obtained PWS iPSCs demonstrated, in all iPSC lines bar one, similar methylation levels compared to the fibroblasts used for reprogramming, including a methylated maternal allele, and an unmethylated paternal allele. The iPSC line with an aberrantly methylated PWS IC was not used for further study.

Yang and colleagues derived iPSCs from the fibroblasts of a diagnosed PWS patient with a balanced translocation of the 15q11-q13 region to chromosome 4 [80]. They were deemed suitable to model PWS in vitro as they maintained characteristics synonymous with the disease, including high DNA methylation levels in the maternal PWS IC and a diminished expression of PWS-associated imprinted genes. These iPSCs were also successfully differentiated into neuronal-like cultures. It was not, however, determined if other functionally relevant genetic or epigenetic aberrations were present in the cultures. Nonetheless, this study emphasizes the usefulness of iPSCs to enhance the understanding of imprinting-related disorders, such as PWS.

Similarly, the generation of AS iPSCs also confirmed the value of such cells to model the disease [81]. Of the three AS iPSC lines used, two contained a large deletion at 15q11-q13, while the third harboured a 2-base pair deletion in *UBE3A*. Differentiated neuronal cultures from control iPSCs established the expected imprinted expression of *UBE3A* with virtually no *UBE3A* expression in the AS-derived cells. Importantly, this study did not determine the status of the methylation imprint at *UBE3A* following reprogramming procedures. 

A landmark earlier study by Chamberlain and colleagues using AS and PWS patient derived iPSCs—genetically conferred rather than caused by an epimutation—utilized DNA methylation analysis, allele-specific PCR, and RNA-FISH and found that copy number variations of the chromosome 15q11-q13 region were maintained through the reprogramming process. It was also observed that DNA methylation at the PWS IC was not altered during reprogramming [82]. This indicates that although substantial epigenetic changes accompany iPSC generation, an intact methylation state at an ICR is faithfully maintained, at least for this imprinted locus. A limitation to this study was that the AS iPSC lines contained sizeable deletions on the maternal chromosomes that consequentially led to the loss of approximately 28 genes. This renders it challenging to identify the specific functions of *UBE3A* in neuronal function and pathogenesis.

In a complementary study, Stanurova and colleagues used iPSCs from an AS patient with a defined 3-base pair deletion in UBE3A [83]. It was reported that, upon the neuronal differentiation of AS iPSCs, the expected imprinted paternal repression of UBE3A and an upregulation of UBE3A-ATS were observed. The cellular models in this study, involving iPSC differentiation into AS and the control mixed neuronal cultures, were therefore demonstrated to successfully replicate the tissue-specific imprinting of UBE3A, leading to reduced expression of UBE3A in the patient-derived cells. Using deep bisulphite amplicon sequencing, it was reported that the differential DNA methylation at a DMR (PWS-SRO) within the PWS IC was maintained through iPSC reprogramming; however, losses and gains of methylation were observed at other regulatory DMRs at the locus. These findings suggest that the appropriate methylation imprints may be vulnerable to iPSC derivation and/or iPSC culture. This might be relevant for conditions associated with ART. 

In general, however, in the studies using iPSCs derived from the AS and PWS patients, iPSCs mostly maintained the methylation status of the PWS-IC. Nonetheless, there have been conflicting reports in which the PWS-IC exhibited hypomethylation in both ‘healthy’ and, importantly, in PWS patient-derived iPSCs [84,85]. Okuno and colleagues observed a reversal of a hypermethylated state of the PWS IC in some PWS iPSC lines derived from one patient [85]. This loss of hypermethylation offers promise for a therapeutic strategy that might reverse the PWS-associated methylation and suggests that patient-derived cells might be susceptible to drug or other treatments that might modulate the DMRs. However, there were clear limitations from this study to be considered, including the fact that cells were derived from only one PWS patient, so no comparison to other patient-derived iPSCs could be made. Furthermore, assessment of the consequences of methylation reversal on transcription was not assessed.

Recently, the first human cell-based model for BWS was also produced using iPSCs derived from a pUPD11 patient, recapitulating the expected transcriptional and epigenetic features of the disease [86]. DNA methylation analysis of the iPSC lines revealed the proper maintenance of the expected methylation at pUPD11 regions. These iPSCs therefore provide a means to elucidate the imprint regulation in BWS including after successful differentiation into hepatocytes. This study, from a patient with mosaicism, derived iPSCs from different fibroblast samples, enabling the use of a non-pUPD11 iPSC line as an isogenic control. It was also demonstrated that the BWS iPSC lines used displayed the proper parent-of-origin methylation status at IC1 and IC2, which were maintained through reprogramming and in culture. It would be of interest for future studies to examine the effects of somatic tissue reprogramming at the IC1 and IC2 DMRs from BWS patients harbouring epimutations, such as a GOM at the maternal *H19/IGF2:IG-DMR*DMR. It could then be determined if such epigenetic alterations are maintained or corrected during reprogramming and will allow for a more comprehensive assessment of the suitability of iPSCs as models for this disorder.

Based on current evidence, the methylated status of ICRs are mostly faithfully recapitulated from imprinting patient-derived iPSCs, suggesting that the reprogramming procedures replicate post-fertilization maintenance of DNA methylation, rather than the germline epigenetic erasure. iPSCs represent a promising modelling strategy for IDs and future studies can consolidate this by investigating whether the methylated status of ID patients with epimutations as a causative factor is accurately reproduced in patient-derived iPSCs.

The combined results of iPSC models of ART-related imprinting disorders show promise and provide an example of the various investigations that are already possible using imprinting patient-derived iPSCs, including the uncovering of phenotypic and mechanistic characteristics underlying the disorders. Future studies can more deeply examine the dynamics of imprinting-related pathologies during tissue specific differentiation, such as the neuronal differentiation of AS iPSCs, for the development of efficient therapies. A current drawback of the use of iPSC models is a variability in the differentiation efficiency amongst iPSC cell lines [82], which could hinder comparisons between studies. Importantly, while much effort is required to understand the effects of reprogramming on the epigenetic landscape of iPSCs derived from patients with IDs if they are to be used for reliable modelling, surprisingly little is known about the stability or vulnerability of normal and abnormal imprints during the iPSC rederivation process in these patient-derived cells. Such data could provide novel insights into the properties of the germline imprint during stem cell reprogramming in vitro, and during the dynamic epigenetic events associated with preimplantation development in ART-associated culture conditions.

## 6. The Effects of Reprogramming on Methylation Status in Normal iPSCs 

To comprehensively assess the potential of iPSCs for modelling imprinting disorders, the effects that reprogramming procedures exert on the normal epigenome must be better understood. Studying the DNA methylation alterations that occur and that induce loss of imprinting (LOI) during iPSC reprogramming could offer valuable insight into the vulnerabilities of imprinted loci in ART-associated imprinting disorders. The epigenetic resetting that occurs during in vitro reprogramming of iPSCs features global DNA demethylation, which is also observed during the reprogramming events in the early embryo and germ line during mammalian development [87]. iPSCs have been reported to harbour epigenetic modifications and genetic deletions due to reprogramming, and genomic imprinting is especially sensitive to reprogramming processes [88,89,90,91,92]. 

A 2014 study demonstrated that iPSC reprogramming with the classic reprogramming factors (Oct3/4, Klf4, c-Myc, Sox2) resulted in the generation of iPSCs that displayed a deviating methylation profile compared to ESCs and retained a somatic cell ‘memory’ of methylation status [93], a phenomenon also observed in a recent study using BWS iPSCs [86]. It has been previously shown that the degree of methylation changes in iPSCs compared to the donor somatic cell is dependent upon the reprogramming efficiency and there are ongoing efforts to increase the efficiency of the reprogramming procedures [94,95].

During reprogramming, iPSC DMRs are obtained in the reprogrammed iPSCs [96,97]. These DMRs are primarily associated with genes and CpG islands and seem to be representative both of the ‘memory’ of the somatic cell methylome and of iPSC-specific DNA methylation signatures. Interestingly, independent iPSC lines have been found to harbour common iPSC-specific DMRs, suggesting an inherent vulnerability of particular loci to the altered methylation obtained during reprogramming procedures. Indeed, genomic imprinting is facilitated by the formation of DMRs at specific genomic loci in gametes [98]. Thus, focused investigations into the susceptibilities of DMRs to aberrant methylation in iPSCs have the potential to unlock further insight into the increased risk of LOI associated with ART and imprinting disorders through the comparison of patterns of vulnerability in reprogrammed iPSCs and LOI in ART patients.

Several factors can influence the variability and status of DMRs in iPSCs, including the genetic background of donor cells [99], the culture conditions [96,100], the method of derivation [93], age of the donated somatic cells [101,102], and the passage number [103]. Given that many imprinted genes are dosage-sensitive regulators of cell proliferation, cell selection within the cultures is likely to contribute to DMR status in culture. Interestingly though, it has been demonstrated that the continued passaging of iPSCs can reduce the divergence of methylation patterns between iPSCs and hESCs [104,105]; however, extended passaging can also result in selection favouring growth-related changes and epigenetic aberrations [106]. For example, the aberrant biallelic expression of the paternally expressed mitogenic *IGF2* gene, is implicated in the phenotypic overgrowth typically presented in BWS [34].

## 7. Imprinting Status of iPSCs 

hPSCs derived via reprogramming methods (iPSCs and ntES) are reportedly more vulnerable to LOI in comparison to hESCs, while some imprinted loci are more susceptible to LOI than others [91,92,93,107,108]. One study identified hypermethylation at the *Dlk1/Dio3*-imprinted region in mouse iPSCs, which led to the improper expression of genes situated within this imprinted locus in these cells, such as *Gtl2* [109]. Therefore, it is suggested that the reprogramming process itself is implicated in the decreased stability of imprinting in reprogrammed cells. Conversely there have been demonstrations that LOI is a rare event in iPSCs [110].

Indeed, several reports that the dynamic addition of de novo methylation marks and their erasure during iPSC culture does not apply to imprinted loci suggests that imprints may be less susceptible to perturbations associated with reprogramming in culture [108,111,112,113]. This implies a distinctive means of regulation of imprinted loci in these cells and that may reflect the in vivo protection that imprints undergo in the periconcep-tional period so that the epigenetic memory of parental origin is preserved. The imprinting status of iPSCs, whether it includes LOI or not, is reportedly maintained during prolonged periods of culture [92,110], and interestingly aberrant imprinting patterns endure throughout differentiation into diverse cellular lineages, just like in vivo. The induction of LOI in iPSCs and maintenance of the imprinting status following lineage-specific differ-entiation are functionally relevant when considering the use of iPSCs for cellular regener-ation therapies and some disease modelling.

Although there are inconsistencies in the literature, findings imply that imprinted DMRs can be susceptible to iPSC reprogramming procedures although their status is maintained during culture [114]. Since several imprinted genes are located within clusters modulated by a unifying germline DMR, an aberration/deletion to a single DMR resulting in LOI can result in loss of expression or biallelic expression of multiple genes in theses clusters [2,115]. It has been suggested that the LOI during reprogramming is mediated by Ten-eleven translocation methylcytosine dioxygenase (TET) proteins, which are cata-lysts in the oxidation of 5-mC to 5-hmC. During the production of iPSCs there is a signifi-cant increase in 5-hmC, similarly observed during in vivo reprogramming. This is proba-bly due to higher expression of TET1 and TET2 proteins, the depletion of which leads to a lower efficiency of iPSC reprogramming, suggesting a prominent role for TETs in the pro-cess [116]. In [117], Bermejo-Álvarez and colleagues proposed that TETs are responsible for LOI at the H19 locus during reprogramming, as is observed in hESCs; however, it is postulated that there may be other, more complex events influencing the regulation of DNA methylation patterns during reprogramming [87].

Varying rates of LOI amongst different hPSCs are accompanied by biallelic expres-sion/repression in the affected imprinted domains. Evidence from several iPSC lines has demonstrated that there is a set of imprinted genes that frequently exhibit biallelic expres-sion, including IGF2, H19, PEG3, PEG10, MEG3, and MEST [84,91,93,107]. In a large-scale analysis of LOI in various iPSC lines, Bar and colleagues [92] identified the most com-mon imprinted loci to display LOI were, in no particular order, MEG3/DLK1 (chr14q32.2), H19/IGF2:IG-DMR (chr11p15.5), and Zdbf2/GPR1 (chr2q33.3). Interestingly, these im-printed regions are all under the control of a paternally methylated DMR, though the pa-ternal mark at Zdbf2 is likely a somatic DMR and is therefore less likely to be affected by ART procedures [118]. Currently, 23 DMRs have been identified in the germ line, and in only three of these DMRs is DNA methylation established on the paternally inherited chromosome; the rest are methylated in the maternal germline [119]. Previous research utilizing iPSCs suggests that imprinted genes under the regulation of paternally methyl-ated DMRs are at a higher vulnerability to LOI than those under the control of maternally methylated DMRs [92,120]. The observed elevated susceptibility of genes under the regu-lation of paternal DMRs to LOI is evident in iPSCs, which implies that paternally methyl-ated regions are more vulnerable to alterations during reprogramming procedures. 

This vulnerability of various imprinted genes can potentially be explained by the re-sistance of the imprinted genes to methylation erasure during the pre-implantation stages of development [120]. The DNA methylation status at the DMRs must be maintained in order to preserve the memory of parental origin and during early developmental stages; ZFP57 and ZNF445 are both modifiers that are essential for the maintenance of imprints during genome-wide methylation erasure.

Zygotic ZFP57 has been demonstrated to be crucial for the maintenance of DNA methylation at some imprinted regions during iPSC derivation, such as *DLK1*/*DIO3* and *SNRPN* [121]. However, ZFP57 was not required for the maintenance of methylation imprints of other regions such as *PEG1* and *PEG3*, which is now probably explained by the recent discoveries of the role of ZNF445 [52]. Aberrant expression of these ZFPs could therefore contribute to the altered methylation state at imprints in cultured PSCs. In mouse the expression of ZFP57 is associated with pluripotency, with high expression detected in oocytes and the early embryo, and a gradual decrease in expression as lineage-specific differentiation progresses. ZFP57 expression is then undetectable in somatic cells [122,123]. This could offer an explanation to the increased vulnerability of iPSCs to aberrant imprinting compared to ESCs, as iPSCs are derived from somatic cells that have a lower protective capacity over their imprinted regions. It is also a possibility that faulty regulation of DNMTs and TET proteins in culture could result in alterations to the ZFP binding sites; demethylation of the ZFP57 binding motif will render it unrecognisable by ZFP57, meaning KAP1 and DNMTs will not be recruited for DNA methylation maintenance, or to replace lost methylation [124].

Altered expression of ZFPs could explain imprinting aberrations in iPSCs and may also play a role in the imprinting defects observed in ART patients that lead to increased incidence of imprinting disorders. Further investigations into the effects of various ART procedures on the regulation of ZFP57 and ZNF445 could uncover more information on the increase in imprinting disorders observed in ART patients. It could also be interesting for future studies to monitor ZFP regulation in stem cell cultures to determine if they con-tribute to the increased susceptibility of iPSCs to LOI.

Alterations at imprinted loci in iPSCs are consistent with findings from studies investigating the methylation profiles of the DMRs of imprinted genes in ART-conceived children. Barbaret and colleagues [125] reported a significant decrease in the methylation levels at the *H19/IGF2:IG*-DMR, and a significant increase in the methylation of the *PEG3* DMR in children conceived via ART procedures (IVF/ICSI) compared to naturally conceived children. Results from this study also suggests that *MEG3* DMRs are vulnerable to ART. The biallelic expression *MEG3* has also been frequently reported in iPSCs.

Another study found that human placental DNA methylation levels at the *H19/IGF2:IG-DMR* and *kcnq1ot1* imprinted loci were reduced in IVF/ICSI, and a decrease in methylation level at *H19/IGF2:IG-DMR* was observed in placentas after IVF compared to ICSI [126]. A limitation to these studies, however, was their inability to completely distinguish the contributions of ART and parental infertility to the altered DMRs. Considering similar alterations are observed at imprinted loci in iPSC culture, future studies that unearth the mechanisms of LOI and methylation changes in iPSCs could provide further mechanistic information regarding the alterations observed at DMRs associated with imprinted genes, and further clarify the association between ART and IDs. 

## 8. Conclusions

Until large cohort studies using thorough and standardized analysis methods are conducted on the ART population, model systems will remain the gold standard for understanding how ART influences the epigenome. Mouse models, which have been used to pioneer the field of imprinting, will continue to be fundamental tools in demystifying the relationship between ART and IDs. However, due to deviations in genomic imprint regulation and preimplantation development between rodents and humans, human studies are necessary for mechanistic studies into genomic imprinting and related disorders. The use of human stem cells bridges the gap between clinical data and animal models.

Most current studies reporting the methylation of ICRs in ID patient-derived iPSCs report an accurate recapitulation of methylated status compared to their somatic cells of origin. This suggests that patient-derived iPSCs could be a good model for epimutation induced IDs. 

The altered methylation and LOI observed in normal iPSCs following reprogramming mirror many of the same defects found in embryos after ART procedures. Understanding the exact mechanisms by which imprinted gene regulation is lost in iPSCs can in turn clarify the epigenetic mechanisms underlying IDs and how they are affected during ART procedures. Culture conditions and cell handling can then be better optimized to reduce stress on vulnerable loci.

The first ART-conceived human precedes the discovery of genomic imprinting in mammals by several years. This is just one example of how medical technologies often outpace our basic understanding of biological processes. Furthermore, this underscores the importance of continuously reassessing and improving existing methods. Increased safety and reduced epigenetic abnormalities from ART procedures can be achieved through the knowledge gained from animal and stem cell-based studies, which can ultimately lead to better health outcomes for ART patients.

## Figures and Tables

**Table 1:** DNA methylation and expression alterations of the imprinted genes implicated in IDs after ART procedures in mice.

Procedure	Imprinted Gene	Reported Alteration	ID Associated with Imprinted Region	References
Ex vivo embryo culture	*H19*	LOM and biallelic expression LOM at ICR	SRS	[62,63] [56,64]
IVF	*H19* *H19*	Aberrant imprint methylation resulting in biallelic expression rather than expression solely from maternal allele. Aberrant methylation patterns at ICR LOM at ICR GOM at maternal ICR	SRS BWS	[55] [65] [64] [55]
Vitrification	*Grb10* *KvDMR1*	Reduced expression accompanied by downregulation of methylation. Reduced methylation does not explain altered expression. GOM in fetuses compared to in vitro culture samples	SRS BWS	[66] [54]
ICSI	*Snrpn* *Peg3* *H19*	LOM at maternal DMR and aberrant expression LOM at maternal DMR and aberrant expression LOM at ICR	PWS / SRS	[67] [64]
Superovulation	*H19* *Snrpn* *Kcnq1ot1* *Gbr10*	Aberrant expression Increased expression LOM at paternal allele LOM at maternal ICR LOM at DMR LOM at maternal ICR GOM at CGI1 and decreased expression	SRS PWS BWS SRS	[65] [45] [44] [44] [68] [44] [45]
In vitro follicle culture	*H19* *Snrpn* *Mest*	LOM at DMR LOM at DMR LOM at DMR	SRS PWSSRS	[68] [68] [68]

## Data Availability

Not applicable.

## Abbreviations

ART	Assisted reproductive technology
IVF	In vitro fertilization
ICSI	Intracytoplasmic sperm injection
hPSCs	Human pluripotent stem cells
hESCs	Human embryonic stem cells
ICM	Inner cell mass
iPSCs	Induced pluripotent stem cells
ntESCs	Nuclear transfer embryonic stem cells
AS	Angelman syndrome
PWS	Prader–Willi syndrome
LOI	Loss of imprinting
lncRNA	Long non-coding RNA
BWS	Beckwith–Wiedemann syndrome
DMR	Differentially methylated region
SRS	Silver–Russell syndrome
ICR	Imprinting coding region
TET	Ten-eleven translocation methylcytosine dioxygenases
5mC	5-Methylcytosine
5hmCe	5-Hydroxymethylcytosine
ZFP	Zinc finger protein
DNMT	DNA methyltransferase
KAP1	KRAB-associated protein 1
mESC	mouse embryonic stem cells
LOM	Loss of methylation
GOM	Gain of methylation
SNP	Single nucleotide polymorphism
ID	Imprinting disorder
